# Supplementary figures and images for: Antibacterial activity and mechanism of sanguinarine against Providencia rettgeri in vitro
Source: PeerJ. 2020 Aug 11;8:e9543. doi: 10.7717/peerj.9543 (PMC7427548; doi:10.7717/peerj.9543)

0MIC

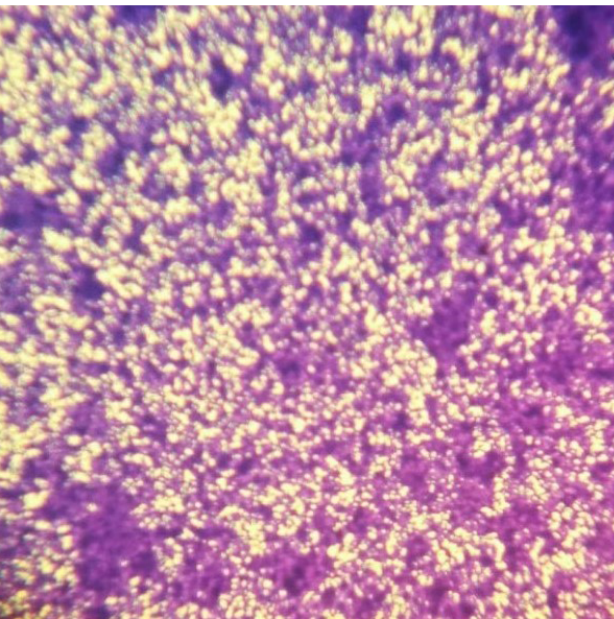

1/16MIC

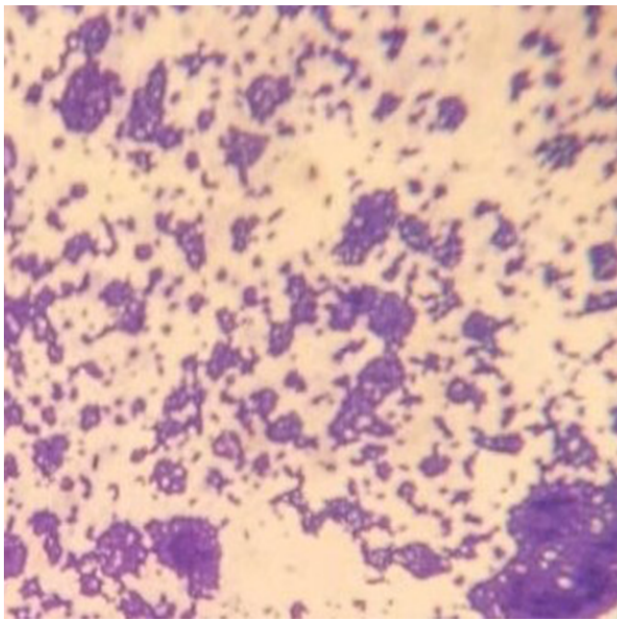

1/8MIC

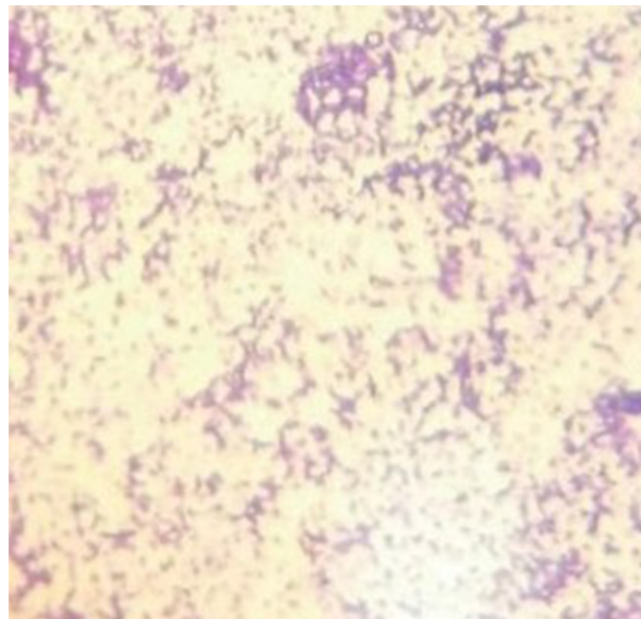

1/4MIC

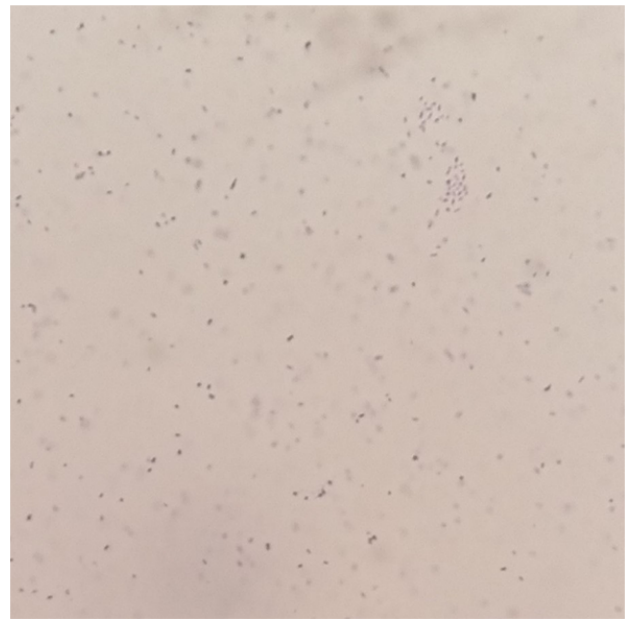

Supplement: Supplemental Information 3 — The effect of different concentration of SAG on PR was observed by crystal violet. SAG concentrations were 0, 1/16, 1/8 and 1/4 MIC respectively. [file peerj-08-9543-s003.pdf]
